# Nicotinamide Administration Improves Remyelination after Stroke

**DOI:** 10.1155/2017/7019803

**Published:** 2017-06-01

**Authors:** Congxiao Wang, Yi Zhang, Jie Ding, Zhen Zhao, Cheng Qian, Ying Luan, Gao-Jun Teng

**Affiliations:** Jiangsu Key Laboratory of Molecular and Functional Imaging, Department of Radiology, Zhongda Hospital, Medical School of Southeast University, Nanjing 210009, China

## Abstract

**Aims:**

Stroke is a leading cause of morbidity and mortality. This study aimed to determine whether nicotinamide administration could improve remyelination after stroke and reveal the underlying mechanism.

**Methods:**

Adult male C57BL/6J mice were intraperitoneally (i.p.) administered with nicotinamide (200 mg/kg, daily) or saline after stroke induced by photothrombotic occlusion of the middle cerebral artery. FK866 (3 mg/kg, daily, *bis in die*), an inhibitor of NAMPT, and ANA-12 (0.5 mg/kg, daily), an antagonist of tropomyosin-related kinase B (TrkB), were administered intraperitoneally 1 h before nicotinamide administration. Functional recovery, MRI, and histological assessment were performed after stroke at different time points.

**Results:**

The nicotinamide-treated mice showed significantly lower infarct area 7 d after stroke induction and significantly higher fractional anisotropy (FA) in the ipsilesional internal capsule (IC) 14 d after stroke induction than the other groups. Higher levels of NAD^+^, BDNF, and remyelination markers were observed in the nicotinamide-treated group. FK866 administration reduced NAD^+^ and BDNF levels in the nicotinamide-treated group. ANA-12 administration impaired the recovery from stroke with no effect on NAD^+^ and BDNF levels. Furthermore, lesser functional deficits were observed in the nicotinamide-treated group than in the control group.

**Conclusions:**

Nicotinamide administration improves remyelination after stroke via the NAD^+^/BDNF/TrkB pathway.

## 1. Introduction

Ischemic stroke is a leading cause of mortality and morbidity [1]. To date, recombinant tissue plasminogen activator (rtPA) is the only approved drug for the treatment of acute ischemic stroke with a 4.5 h window for therapeutic intervention after ischemic stroke onset [2–6]. However, very few patients with acute ischemic stroke receive this therapy. Moreover, as many as half of the patients with ischemic stroke receiving rtPA treatment survive and suffer long-term disability. Therefore, there is considerable interest in developing new treatment modalities for stroke, with focus on restorative therapeutics [7].

Over the past decades, the majority of studies have focused on the insults suffered by the gray matter after stroke, while less attention has been paid to white matter injuries [8]. However, white matter constitutes about 50% of the brain volume in humans, and clinical data suggest that ischemic stroke in most cases damages both white matter and gray matter [9]. The white matter, constituted of axons and glial cells including oligodendrocytes, astrocytes, and microglia, is vulnerable to ischemic insults and can be considerably damaged also by brief focal ischemic episodes. Notably, white matter injury is the major cause of functional disability after ischemic stroke [9–11]. In the central nervous system, information is rapidly transmitted between different brain regions along axons, which are enveloped in a multilayered membrane sheath formed by oligodendrocytes that increase the conduction speed of action potentials via accelerating impulse conduction [12, 13]. Myelin sheath has a vital role for cell interaction involving information exchange between oligodendrocytes and axons; it provides nutrition support for neurons and is important for the motor, sensory, and cognitive functions of the nervous system [13, 14]. Damage to oligodendrocytes caused by ischemia causes loss of action potential propagation through myelinated axons also when the neuronal integrity is preserved [15]. Therefore, we believe that it is important to develop an effective therapy to promote remyelination after stroke in consideration of the important role of myelin in the functioning of the nervous system and the consequences of its damage after stroke insult.

Nicotinamide (NAM) is the amide derivative of nicotinic acid (also known as vitamin B3 or niacin). NAM, a precursor of nicotinamide adenine dinucleotide (NAD^+^), is converted into NAD^+^ by nicotinamide phosphoribosyltransferase (NAMPT), a rate-limiting enzyme in NAD^+^ biosynthesis in vivo [16]. Despite structural similarities, NAM has different functions and clinical uses from nicotinic acid. NAM is an approved drug for medical use and shows a good safety profile at high doses. Moreover, the vasodilatory side effects of nicotinic acid such as hypotension, headache, and flushing are not observed with NAM [17]. In the recent years, NAM has been used in animal models and clinical trials to assess its efficacy in treating a variety of diseases including type 1 diabetes, acute lung injury, Friedreich's ataxia, skin cancer chemoprevention, Parkinson's disease (PD), Huntington's disease (HD), and ischemic stroke [18–22]. To date, the majority of the studies have investigated the therapeutic effects of NAM on the acute phase of ischemic stroke, and different mechanisms involved in its protective effect have been described [19] yet overlook the possible effect of long-term NAM administration on remyelination after stroke. Our study aims to investigate whether long-term NAM administration could be used as a restorative therapy to promote the remyelination after stroke and to reveal the underlying mechanism.

## 2. Methods

### 2.1. Photothrombotic Middle Cerebral Artery Ischemic Stroke Model

All animal experiments in this study were approved by the Institutional Animal Care and Use Committee (IACUC) of Southeast University (approval ID: SYXK-2010.4987). Adult male C56BL/6J mice (20.0–25.0 g, 8–10 wk, Comparative Medicine Centre of Yangzhou University [SCXK (SU) 2012-0004], Yangzhou, China) were used in this study. Photothrombotic ischemic stroke was induced by a well-established previously reported procedure [23, 24]. Briefly, mice were anesthetized with pentobarbital (50 mg/kg; 1% in sterile saline), and body temperature was maintained at ~37°C with a heating pad. An incision was made between the right orbit and the external auditory canal to separate the temporalis muscle from the dura mater, and the proximal section of the middle cerebral artery was exposed. The photosensitizer Rose Bengal (0.025 mmol/kg, Sigma-Aldrich, Shanghai, China) was injected intravenously, and after 2 min, a laser beam (diameter: 0.1 mm, wavelength: 532 nm, Shanghai Laser & Optics Century Co. Ltd., Shanghai, China) was focused on it. The skin was then sutured and mice were returned to the cages to regain consciousness.

### 2.2. Drug Administration and Experimental Groups

Each experimental group included 16 mice. Four experimental groups were identified as follows: Group 1, mice were treated with NAM alone; Group 2, mice were treated with NAM and ANA-12; Group 3, mice were treated with NAM and FK866; and Group 4, mice were treated with saline (control group). NAM (200 mg/kg, Sigma-Aldrich, Shanghai, China) was injected intraperitoneally (i.p.) daily for 14 d; the first injection occurred 1 h after stroke induction. ANA-12 (0.5 mg/kg, Sigma-Aldrich, Shanghai, China), a selective antagonist of tropomyosin receptor kinase B (TrkB), was injected intraperitoneally 1 h before NAM injection. FK866 (3 mg/kg, Selleck Chemicals, Shanghai, China), an antagonist of nicotinamide phosphoribosyltransferase (NAMPT), was intraperitoneally injected twice a day for 14 d, the first dose administered 1 h before NAM injection. The same amount of volume of saline solution was intraperitoneally administered to the control group.

### 2.3. Magnetic Resonance Imaging

Magnetic resonance imaging (MRI) was performed using a 7 T small animal magnetic resonance system (PharmaScan; Bruker, Ettlingen, Germany). Mice were anesthetized with 1% isoflurane (Shandong Keyuan Pharmaceutical Co. Ltd., Shandong, China), heart rate maintained at ~100 bpm. At 24 h and 7 d poststroke, turbo spin-echo sequence T2-weighted images (T2WI) were collected with the following set of parameters: field of view = 2 cm × 2 cm, slice thickness = 1 mm, slices = 15, interslice distance = 1 mm, repetition time = 3 s, averages = 1, matrix size = 256 × 256, flip angle = 180°, and total scan time for image acquisition: 1 min 20s. For diffusion tensor imaging (DTI), an echo-planar imaging (EPI) sequence was used to acquire 30 distinct diffusion directions. Five reference images were obtained at 14 d with the following set of parameters: field of view = 2 cm, slice thickness = 0.6 mm, slices = 20, matrix size = 128 × 128, repetition time = 5 s, average = 2, and total scan time for DTI: 23 min 20s. The infarct volume was calculated with ImageJ software (National Institutes of Health, Bethesda, MD) using the equation: infarct sizes %  = ∑ {infarct area − (ipsilateral hemisphere − contralateral hemisphere)} × 100/∑(contralateral hemisphere). Fractional anisotropy (FA) was measured from the tensor map in the ipsilateral internal capsule (IC) with ParaVision 5.0 software (Bruker, Ettlingen, Germany). Fiber tracking was carried out using TrackVis (Version 0.5.2.1, Center for Biomedical Imaging, Department of Radiology, Massachusetts General Hospital) software and Diffusion Toolkit (version 0.6.2.1) in the ipsilateral internal capsule (IC) of the brain.

### 2.4. Immunohistochemistry

Mice brains were extracted at 7 d and 14 d and fixed in 4% paraformaldehyde overnight at 4°C. They were subsequently immersed in 30% sucrose-containing tubes until they sunk to the bottom, before being transferred onto a freezing microtome (CM1950, Leica Biosystems Nussloch GmbH, Nussloch, Germany) to obtain slices of 10 *μ*m thickness. To confirm the expression of O4, brain-derived neurotrophic factor (BNDF), and myelin basic protein (MBP), immunofluorescence staining was carried out. Brain sections were rehydrated using phosphate-buffered saline (PBS) for 10 min before being blocked with PBT buffer (3% BSA and 0.1% Triton X-100 in PBS) for 1 h at 25°C. Further, brain sections were incubated with rabbit anti-mouse BDNF polyclonal antibody (1 : 500 dilution; Abcam, Cambridge, UK), rabbit anti-mouse MBP antibody (1 : 500 dilution; Abcam, Cambridge, UK), and mouse anti-mouse monoclonal antibody (1 : 100 dilution; Merck Millipore, Shanghai, China) separately overnight at 4°C. After washing three times with PBS, sections were incubated with the secondary antibodies rabbit anti-mouse Alexa Fluor 488 (1 : 500 dilution; Invitrogen, part of Thermo Fisher Scientific, Carlsbad, CA) for O4 staining, goat anti-rabbit Alexa Fluor 546 (1 : 500 dilution; Invitrogen, part of Thermo Fisher Scientific, Carlsbad, CA) for BDNF and MBP staining, for 2 h at 25°C. Finally, after washing for three times with PBS, nuclear DNA was stained with DAPI (SouthernBiotech, Birmingham, AL). Brain sections were observed using a ZEISS microscope and image analysis was carried out using the Image-Pro Plus software (Media Cybernetics, Rockville, MD). All staining was done identically and at the same time on the groups being compared.

### 2.5. Western Blot Analysis

Brain samples were homogenized in RIPA buffer (KeyGEN BioTECH, Nanjing, China) containing 1% protease inhibitor cocktail (Roche Applied Sciences, Penzberg, Germany), and centrifuged at 15,000 rpm for 15 min at 4°C (Centrifuge 5417R; Eppendorf, Hamburg, Germany). Supernatants were transferred into a labeled tube. After quantification using the BCA protein quantification kit (KeyGEN BioTECH, Nanjing, China), proteins were denatured with a loading buffer at 100°C. Equal amounts of proteins were separated using SDS-PAGE and transferred to a PVDF membrane (Bio-Rad Laboratories, Shanghai, China). The PVDF membrane was blocked with 5% milk at 25°C for 1 h before incubation with primary antibody for BDNF (1 : 500 dilution; Abcam, Cambridge, UK), MBP (1 : 1000 dilution, Abcam, Cambridge, UK) and *β*-actin (1 : 5000 dilution; Abcam, Cambridge, UK) overnight at 4°C. After washing with TBST buffer for three times, an HRP-conjugated secondary antibody diluted 1 : 5000 was used to recognize the primary antibody. Finally, the membrane was exposed and protein bands were analyzed using ImageJ software (NIH, Bethesda, MD).

### 2.6. NAD^+^ Estimation

NAD^+^ concentrations were determined using the NAD/NADH Quantification Kit (Sigma-Aldrich, Shanghai, China) in accordance with the manufacturer's instructions. Briefly, brain samples were homogenized with a NADH/NAD extraction buffer in a microcentrifuge tube before being spun for 5 min (14,000 rpm, 4°C) to obtain the NAD/NADH supernatant. To avoid being denatured by enzymes, supernatants were deproteinized by filtering through a 10 kDa cut-off spin filter. For total NADH and NAD (NADtotal) detection, 50 *μ*L of the supernatant were transferred into a 96-well plate in duplicate; for NADH only detection, NAD was decomposed by heating to 60°C for 30 min before 50 *μ*L of the supernatant were also transferred into a plate in duplicate. After addition of 100 *μ*L Master Reaction Mix to each of the wells and incubation for 5 min at room temperature, 10 *μ*L NADH Developer was added into each well and left to incubate for 1 h to 4 h at 25°C. The absorbance was read at 450 nm using a Spectrophotometric multiwell plate reader (Multiskan GO, Thermo Fisher Scientific, Waltham, MA, US). Thereafter, NAD concentrations were calculated in accordance with the following equation: NAD^+^ = NAD^+^_total_ − NADH.

### 2.7. Behavioral Test

To assess the functional recovery after stroke in the four different groups, the modified Neurological Severity Score (mNSS score) and the Foot Fault test were performed at 1 d before and 1 d, 3 d, 7 d, and 14 d after stroke induction [25, 26]. In the Foot Fault test, each mouse was placed on a homemade wire grid with a camera beneath it to record the foot faults. Mice were allowed to freely walk on the wire grid for 5 min. The movie was analyzed with a computer. A foot fault was confirmed if the paw went through the grid hole failing to provide support for the mice. Foot faults were calculated with the following equation: foot  faults% = {(number  of  foot faults)/(total  footsteps)} × 100. The mNSS score includes a composite of motor, sensory, beam balance, reflex absence, and abnormal movement tests as previously reported [25, 26]. The observers analyzing the video and recording the results were blind to the experimental conditions.

### 2.8. Statistics

Data were analyzed with SPSS v.19.0 software (SPSS Inc., Chicago, IL, USA) and are shown as the mean ± standard deviation (SD). One-way analysis of variance (ANOVA) and Bonferroni posttests were used to evaluate differences among the groups; *p* < 0.05 was considered statistically significant.

## 3. Results

### 3.1. NAM Administration Led to Reduced Infarct Size, Increased Fractional Anisotropy Value, and Fiber Counts after Stroke

**Figure 1:**
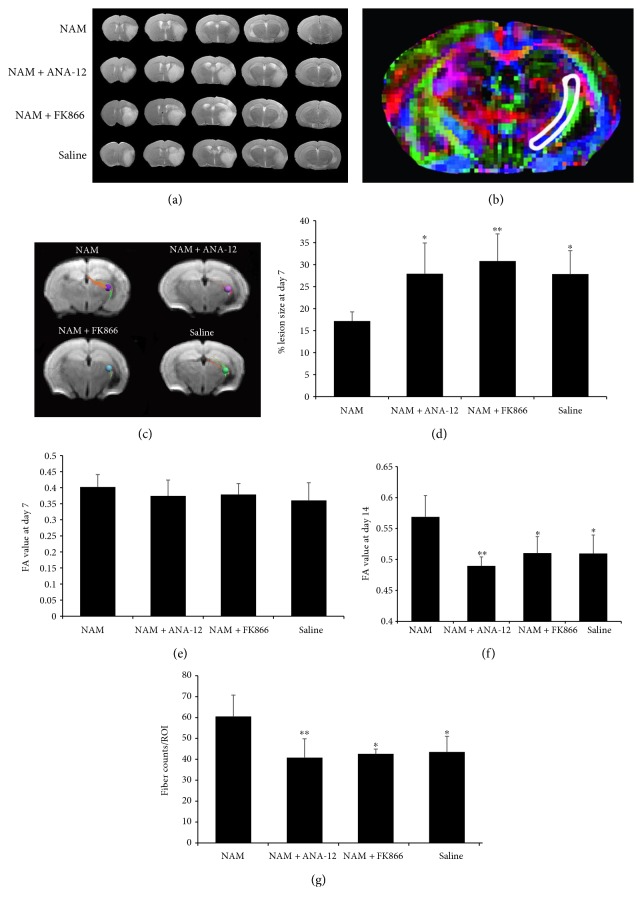
Increased remyelination after NAM administration was observed in vivo with MRI. (a) T2-weighted images showing the reduction of infarct size as observed at 7 d after NAM injection. (b) Representative FA color map of a brain section. The area surrounded by white line was the site of internal capsule selected for FA value determining. (c) Representative photographs of fiber tracking in the region of interest (ROI) of IC from the four groups. ROI radius: 0.45 mm. The directions of fiber tracks are color-coded with red for left–right, blue for superior–inferior, and green for anterior–posterior. (d) Quantification of the infarct size in the four groups. *n* = 6. (e) FA values measured in the IC at 7 d after stroke induction. *n* = 7 for the NAM group; *n* = 6 for the FK866 group; *n* = 5 for the NAM+ANA-12 and saline groups. (f) FA values measured in the IC at 14 d after stroke induction. *n* = 7 for the NAM group; *n* = 6 for the FK866 group; *n* = 5 for the NAM+ANA-12 and saline groups. (g) Quantitative data of fiber counts performed in the ICs of the four groups. *n* = 7 for the NAM group; *n* = 6 for the FK866 group; *n* = 5 for the NAM+ANA-12 and saline groups. Data showed as mean ± SD in all histograms. ^∗^*p* < 0.05, ^∗∗^*p* < 0.01 versus NAM.

In vivo T2WI and DTI were carried out at 7 d and 14 d in the four groups. Reduction of infarct sizes was observed at 7 d of continuous administration of NAM as shown on T2-weighted images (Figures 1(a) and 1(d)). When comparing fractional anisotropy (FA) values in the IC (illustrated in the color map in Figure 1(b)) between the four groups at 7 d after stroke, no significant differences were found (Figure 1(e)). However, at 14 d, the increase in FA values in the NAM group was significantly higher compared to that in the other three groups (Figure 1(f)). In addition, the fiber counts in the IC were also significantly higher in the NAM group compared to those in the other three groups (Figures 1(c) and 1(g)). These results suggested a better recovery of myelination with NAM administration after stroke.

**Figure 2:**
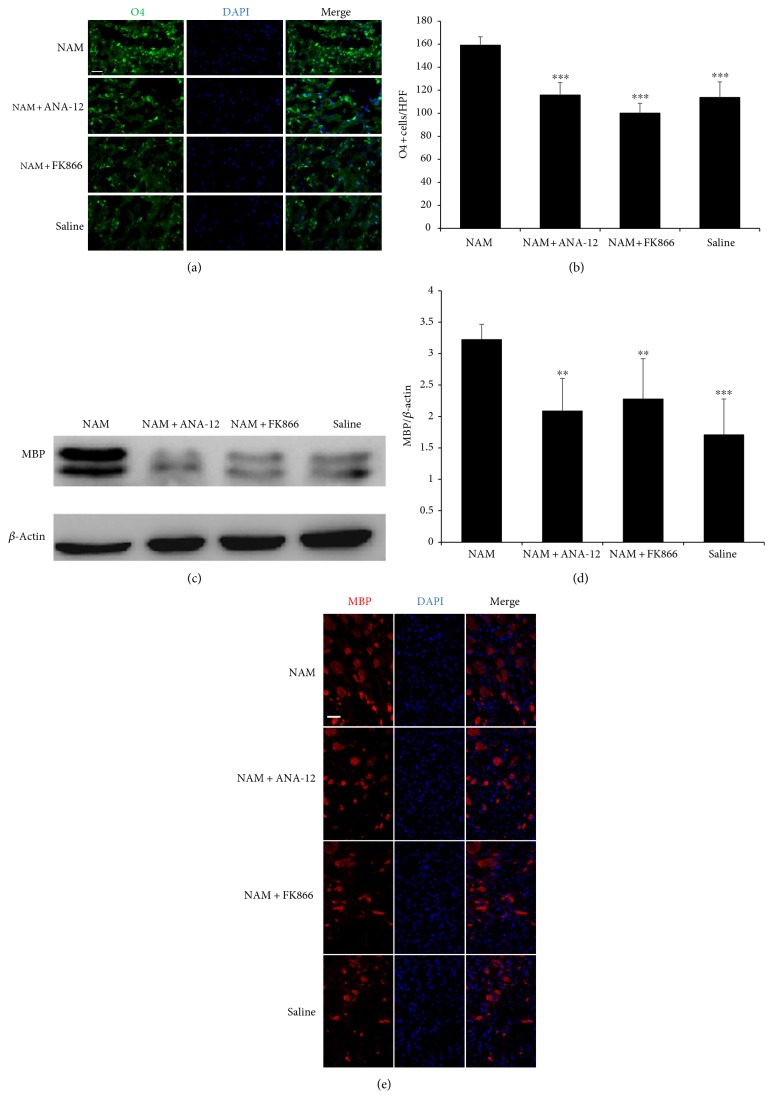
Continuous NAM administration enhanced mature oligodendrocytes generation and remyelination after stroke. Immunofluorescence images (a) and quantitative data (*n* = 4) (b) showing an increase in O4 expression in the NAM group at 7 d after stroke induction. Western blot analysis (c), quantitative data (*n* = 8) (d), and immunofluorescence images (e) showing that MBP expression was higher in the NAM group compared to the other three groups at 14 d after stroke induction. Data showed as mean ± SD in all histograms. ^∗∗^*p* < 0.01, ^∗∗∗^*p* < 0.001 versus NAM. HPF: high power field. Scale bar: 40 *μ*m.

Moreover, analysis of immunofluorescence staining showed that at 7 d after NAM administration, O4 staining, a marker of pro-oligodendrocytes, was higher in the NAM group than that in the other three groups in the subventricular zone (Figures 2(a) and 2(b) and Supplementary Figure 1 available online at https://doi.org/10.1155/2017/7019803), indicating that more mature oligodendrocytes were generated and thus supporting the evidence found with MRI. Western blot analysis and immunofluorescence staining for MBP, a marker for mature oligodendrocytes, showed that MBP expression was higher in the NAM treated group than that in the other three groups in the peri-infarct area at 14 d (Figures 2(c), 2(d), and 2(e) and Supplementary Figure 2), further supporting our MRI results.

### 3.2. Nicotinamide Administration Promoted the Expression of BDNF via Increased NAD^+^ Levels

**Figure 3:**
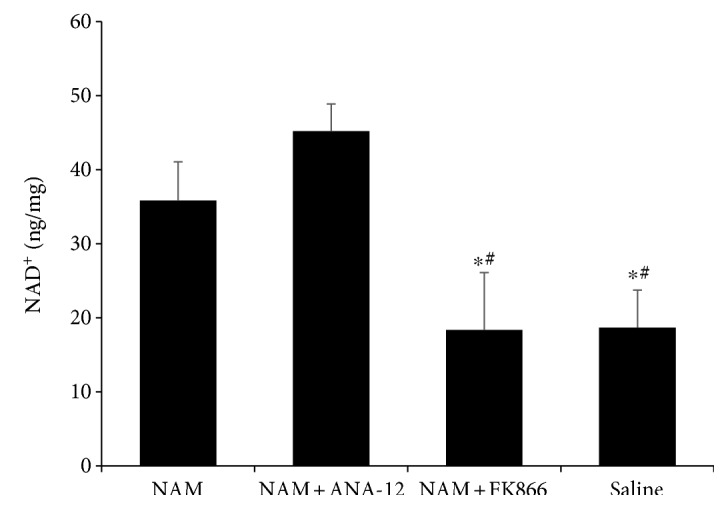
Continuous treatment with NAM induced an increased in NAD^+^ level; FK866 treatment blocked the conversion of NAM into NAD^+^ via inhibition of NAMPT enzyme activity. Data showed as mean ± SD in histograms. *n* = 3; ^∗^*p* < 0.05 versus NAM; ^#^*p* < 0.05 versus NAM+ANA-12.

To verify whether continuous NAM administration could increase NAD^+^ levels in the peri-infarct area of mouse brain long after the stroke, we evaluated the levels of NAD^+^ at 7 d after stroke induction. As expected, NAD^+^ levels were significantly higher in the NAM-treated group than those in the saline-treated group. FK866, an inhibitor of NAMPT, prevented the conversion of NAM into NAD^+^, resulting in a lower NAD^+^ level in the NAM+FK866 group, although mice in this group received continuous NAM administration. In contrast, ANA-12, a TrkB-selective antagonist, had no effect on the NAM-induced increased level of NAD^+^ (Figure 3()).

**Figure 4:**
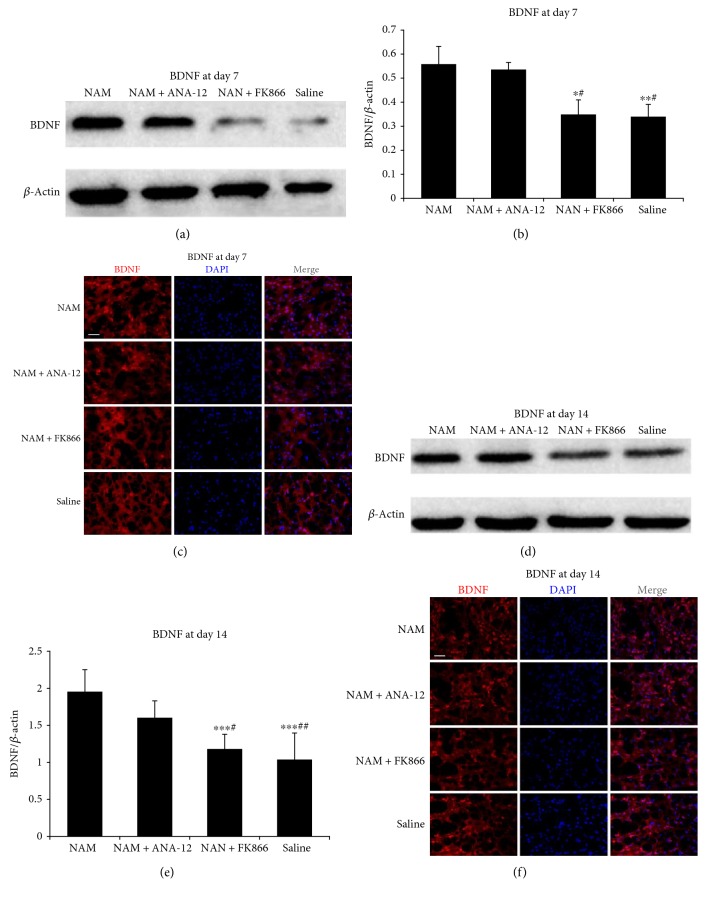
Western blot analysis (a), quantitative data (*n* = 3) (b), and immunofluorescence images (c) showing BDNF expression 7 d after stroke induction. Western blot analysis (d), quantitative data (*n* = 4) (e), and immunofluorescence images (f) showing BDNF expression 14 d after stroke induction. Data showed as mean ± SD in all histograms. ^∗^*p* < 0.05, ^∗∗^*p* < 0.01, ^∗∗∗^*p* < 0.001 versus NAM; ^#^*p* < 0.05, ^##^*p* < 0.01 versus NAM+ANA-12. Scale bar: 40 *μ*m.

To determine whether NAM-induced NAD^+^ elevation affects BDNF expression after stroke, we assessed expression of BDNF in the peri-infarct area of the brain. Our results showed that the expression of BDNF was significantly higher in NAM-treated and NAM+ANA-12-treated groups at 7 d after stroke than BDNF expression level in the NAM+FK866-treated and saline-treated groups (Figures 4(a) and 4(b)), thereby showing a similar trend in NAD^+^ levels detected in the same groups at the same time point. Higher levels of BDNF were observed in the NAM-treated and NAM+ANA-12-treated groups than in the NAM+FK866-treated and saline-treated groups at 14 d after induction of the experimental stroke (Figures 4(d) and 4(e)). These findings were confirmed by immunofluorescence staining (Figures 4(c) and 4(f)).

### 3.3. Elevated BDNF Interacts with TrkB to Promote TrkB Phosphorylation after Stroke

**Figure 5:**
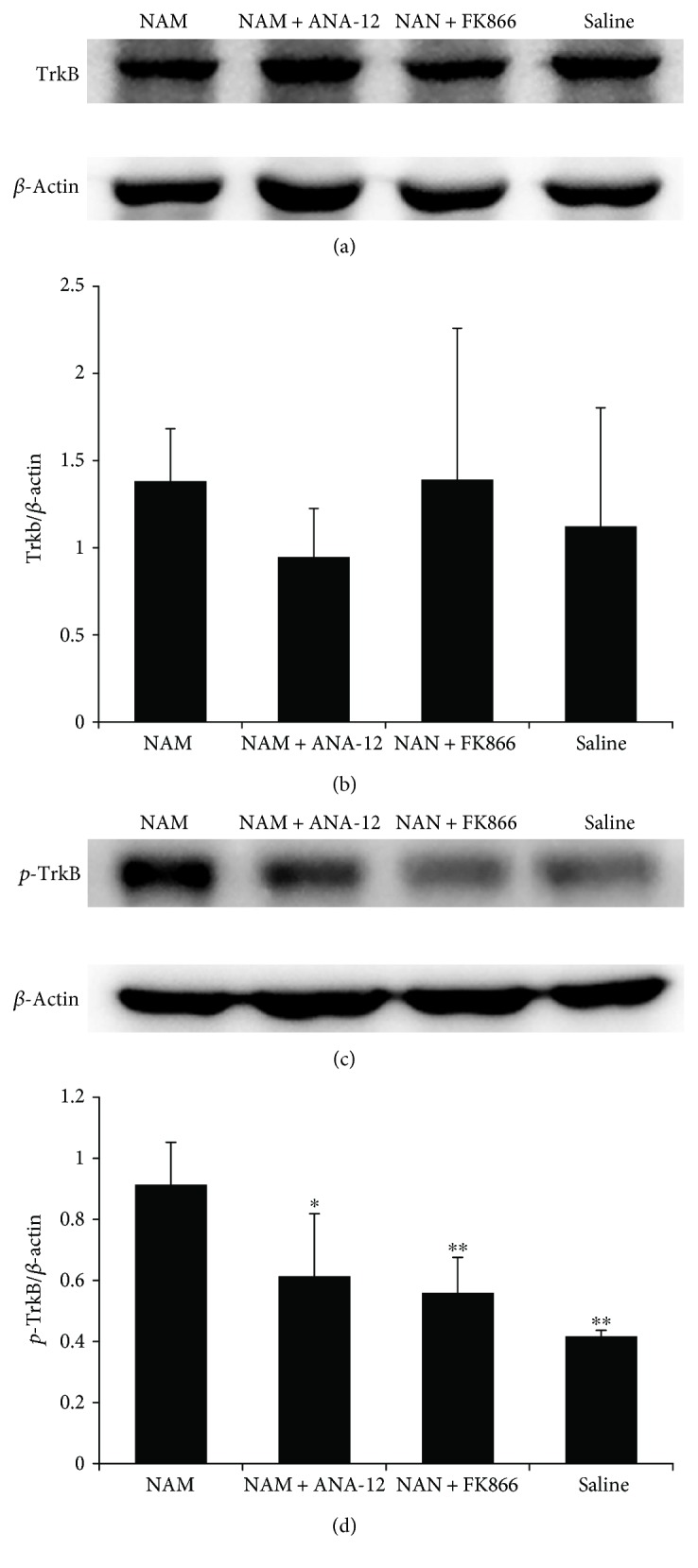
BDNF interacts with TrkB to promote remyelination after stroke. Western blot analysis (a) and quantification data (b) showing no differences between TrkB expressions in the four groups. Western blot analysis (c) and quantification data (d) show that the phosphorylated TrkB (P-TrkB) level was higher in the NAM group compared to the other groups. Data showed as mean ± SD in all histograms. *n* = 4; ^∗^*p* < 0.05, ^∗∗^*p* < 0.01 versus NAM.

To elucidate the interaction between BDNF and its receptor TrkB, we used a selective TrkB antagonist to block BDNF binding. As shown in Figure 5, we found no difference in TrkB expression among the four groups at 14 d. However, phosphorylated TrkB (P-TrkB) expression was significantly higher in the NAM group than in the other three groups. Treatment with ANA-12 blocked NAM-induced phosphorylation of TrkB in the brain.

### 3.4. Nicotinamide Enhanced Functional Recovery after Stroke

**Figure 6:**
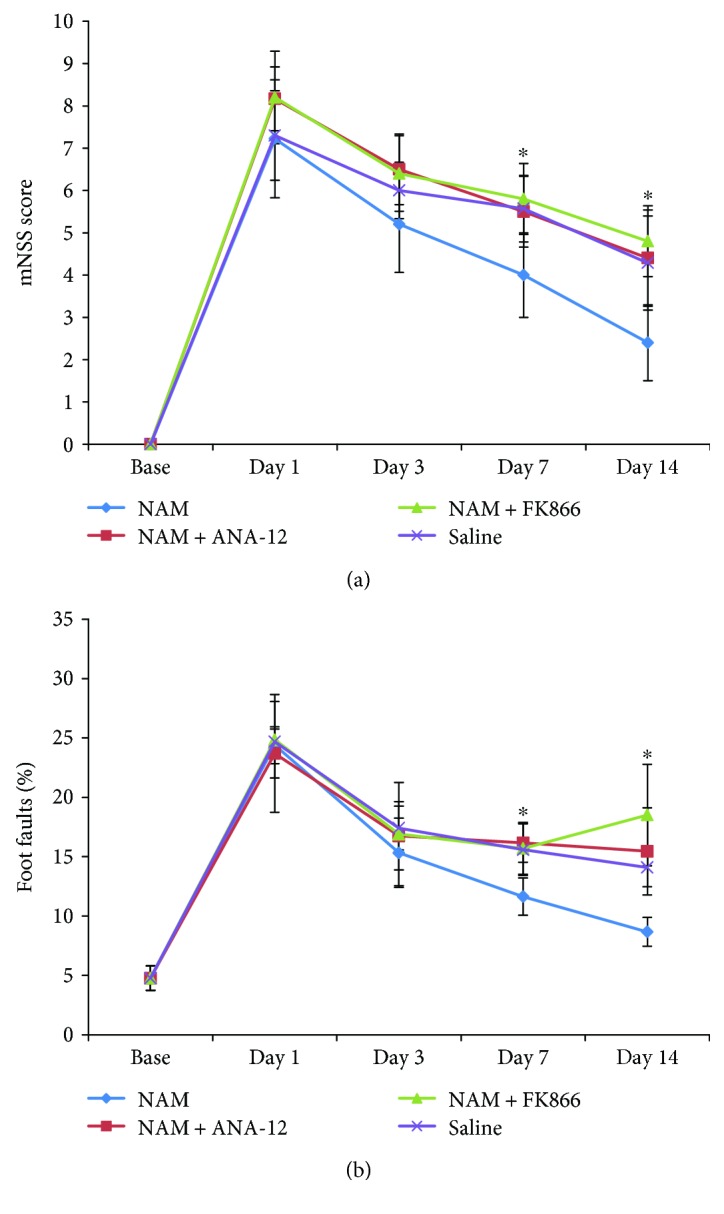
Effect of nicotinamide on functional recovery after stroke. NAM treatment resulted in time-dependent functional recovery after stroke. (a) The modified neurological severity score (mNSS) (*n* = 8 for the NAM and saline groups; *n* = 5 for the NAM+FK866 and NAM+ANA-12 groups) and (b) Foot Fault tests were performed before stroke induction and 1 d, 3 d, 7 d, and 14 d after stroke induction (*n* = 5). Data showed as mean ± SD in all line charts. ^∗^*p* < 0.05 versus NAM.

To determine whether NAM continuous administration had an effect on functional recovery after stroke, mNSS scores, and Foot Fault test were performed before stroke induction and 1 d, 3 d, 7 d, and 14 d after stroke. No significant differences in these test results were observed 1 d and 3 d after stroke induction. However, at 7 d and 14 d after stroke, mice treated with NAM showed a statistically significant reduction of neurological functional deficits compared to those of the other three groups (Figure 6). Mice in the NAM group showed increased motor, sensory, and cognitive functions. Both FK866 and ANA-12 blocked NAM's effect on functional recovery.

## 4. Discussion

Considering the important role of myelin in the brain, it is necessary to emphasize the importance of remyelination poststroke recovery. Assessment of the effects of NAM on ischemic stroke recovery has been carried out extensively. However, since most of the studies have focused on the acute phase of ischemic stroke mostly, they emphasized the protective effects of NAM on cells' death (such as neurons and vascular cells) during ischemic stroke [19, 27, 28]. In our experiments, NAM exerted a positive role in remyelination after stroke. During NAM treatment, a significant increase of O4 expression was observed in the subventricular zone 7 d after experimental stroke induction, indicating that more mature oligodendrocytes were generated, thereby aiding the remyelination process. In addition, 14 d after NAM treatment, more cells in the peri-infarct area showed expression of MBP, a specific maker of mature oligodendrocytes. The results suggested that treatment with NAM induced more pro-oligodendrocytes to transform into mature ones resulting in a better recovery of myelination after stroke. MRI has been extensively used to evaluate therapy efficiency and predict the recovery after ischemic stroke. MRI monitors noninvasively biological processes at a high resolution and in real time. Furthermore, by using MRI, we obtained a series of consecutive images in the same subject, thereby obtaining more detailed information by processing the statistic power of longitudinal studies. MRI also enables repetitive imaging in the same subject. Within the organized glial bundles surrounding the tightly packed and coherently aligned axons, the movement of water molecules perpendicular to the orientation of axons were hindered to a greater extent compared to the movements parallel to the axon orientation, resulting in anisotropic diffusion [29]. It has been reported that myelinated axons could strongly restrict diffusion of water in the white matter [30].

Therefore, we sought to evaluate the recovery of myelination after stroke by analyzing fractional anisotropy (FA) of water diffusion obtained from DTI in vivo with MRI scanning. Moreover, fiber counts were also assessed after reconstruction of DTI to assess the recovery of myelination. We carried out T2WI and DTI with a 7 T small animal magnetic resonance system 7 d and 14 d after experimental stroke induction. A significant decrease of the infarct size was observed in the NAM group; however, no significant differences in the FA values obtained from DTI were observed in the IC 7 d after the stroke. A significant increase of FA values was observed after 14 d in the NAM group, suggesting that myelinated axons increased after NAM administration, reflecting the occurrence of the remyelination process after the stroke. DTI analysis showed an increase in fiber counts in the NAM group. A similar trend was observed for O4 and MBP expressions detected with Western blot analysis and immunofluorescence staining. To our knowledge, this is the first study showing that NAM administration could promote remyelination after stroke. Owing to the important role of myelin in maintaining correct axonal conduction velocity across the brain, functional recovery assessment is useful to evaluate the extent of myelination recovery. Previous studies have confirmed that mNSS and Foot Fault tests are powerful to evaluate the functional recovery after stroke [31, 32]. In this study, NAM treatment induced a significant improvement in functional recovery 7 d and 14 d after stroke compared to the other study groups.

Brain-derived neurotrophic factor (BDNF) is one of the most important trophic factors in the brain. BDNF has been shown to modulate neuronal circuits, promote neurogenesis, increase synaptic plasticity and axon growth, and enhance angiogenesis [33]. BDNF binds to two different receptors: the tropomyosin-related kinase receptor B (TrkB) and the p75 neurotrophin receptor (p75 NTR) [34, 35]. It has been reported that interactions between BDNF and TrkB potentiate CNS myelination during early postnatal development. BDNF promotes oligodendroglia maturation and myelination during development [36]. In vitro and BDNF knockout studies also emphasize the important effects of BDNF on myelination [37, 38]. To investigate whether BDNF played an important role in the remyelination process after NAM treatment in ischemic stroke, we detected the expression of BDNF in the peri-infarct area 7 d and 14 d after stroke induction. Results suggested that NAM enhanced the expression of BDNF after stroke, which correlates with enhanced recovery of myelination. To determine whether BDNF/TrkB pathway plays a role in the remyelination process during stroke recovery, we tested the effect of ANA-12, a selective TrkB antagonist. Our results showed that ANA-12 effectively blocked the phosphorylation of TrkB, and accordingly no better remyelination was observed in the NAM+ANA-12-treated group, despite higher BDNF expression being observed in the NAM-treated and NAM+ANA-12-treated groups. This result confirmed the important role played by BDNF in remyelination after stroke and suggested that NAM administration promotes remyelination via the BDNF/TrkB pathway.

Although our results demonstrated that BDNF/TrkB pathway plays an important role in remyelination during NAM treatment after stroke, it is unknown how NAM enhanced BDNF expression after stroke. NAM is converted into NAD^+^ by NAMPT in vivo via the salvage pathway, the main synthesis pathway of NAD^+^ in mammalian cells [39]. NAD^+^ is associated with aging, metabolism, and neurodegeneration [40]. Lower NAD^+^ levels have been reported to reduce activity-dependent expression of BDNF owing to its effect on the increased methylation of BDNF promoter [41]. In vitro studies showed that administration of NAM could prevent NAD^+^ depletion to protect neurons against damage from excitotoxicity, and this effect was also confirmed in vivo in an acute poststroke phase [42, 43]. However, no previous studies have investigated whether long-term NAM treatment could preserve NAD^+^ at a higher level after stroke. In addition, we also wanted to determine whether increased BDNF expression was due to elevated NAD^+^ after NAM administration. Our results showed that NAD^+^ levels were higher in the NAM-treated group 7 d after stroke induction. When we blocked the conversion of NAM into NAD^+^ via NAMPT inhibition by a selective (FK866), the elevated NAD^+^ level caused by NAM administration was also abolished. This resulted in decreased BDNF expression in the NAM+FK866-treated group. Therefore, our results suggest that NAM administration enhanced BDNF expression via increased NAD^+^ level after stroke induction. Increased BDNF levels facilitated enhanced recovery of myelination after stroke via activation of TrkB receptor. The effect of NAM on remyelination was blocked by either inhibiting the activity of NAMPT to prevent NAM conversion into NAD^+^ or blocking BDNF binding to TrkB after NAM treatment. Lacking of the two other control groups which were ANA-12 or FK866 treatment alone could be one of the limitations in this study. With them, specificity of the two inhibitors (ANA-12 and FK866) on NAM actions could have exhibited better.

## 5. Conclusions

We showed that NAM administration could promote remyelination after stroke via a NAD^+^/BDNF/TrkB pathway. Our studies supplied a new restorative therapeutic strategy for the treatment of ischemic stroke.

## Supplementary Material

Supplementary figure 1. Sampling regions of O4 detection in immunofluorescence assay was indicated with blue boxes. Supplementary figure 2. Sampling region of MBP detection in immunofluorescence assay was indicated with blue box.
