# Supplementary material for: Nicotinamide Administration Improves Remyelination after Stroke
Source: Neural Plast. 2017 Jun 1;2017:7019803. doi: 10.1155/2017/7019803 (PMC5471593; doi:10.1155/2017/7019803)

Supplementary figure 1. Sampling regions of O4 detection in immunofluorescence assay was indicated with blue boxes.

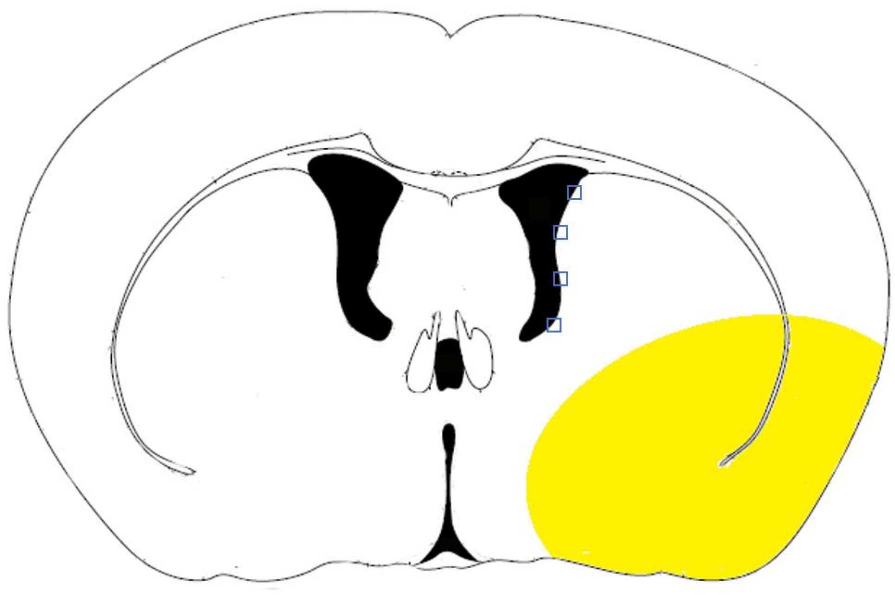

Supplementary figure 2. Sampling region of MBP detection in immunofluorescence assay was indicated with blue box.

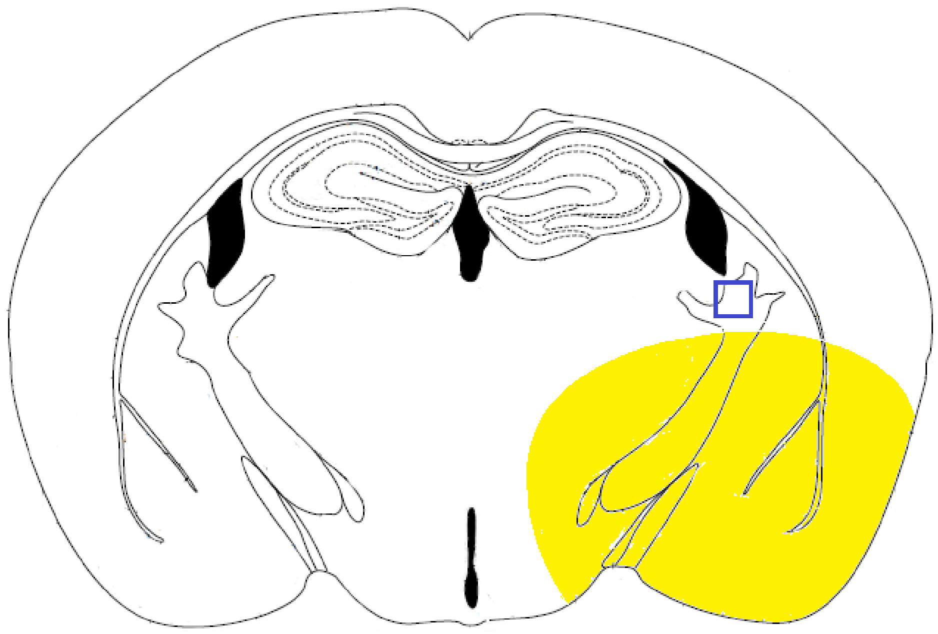

Supplement: Supplementary file 1 — Supplementary figure 1. Sampling regions of O4 detection in immunofluorescence assay was indicated with blue boxes. Supplementary figure 2. Sampling region of MBP detection in immunofluorescence assay was indicated with blue box. [file 7019803.f1.pdf]
