# Supplementary material for: Nicotinamide Administration Improves Remyelination after Stroke
Source: Neural Plast. 2017 Jun 1;2017:7019803. doi: 10.1155/2017/7019803 (PMC5471593; doi:10.1155/2017/7019803)

*Supplementary figure 1. Sampling regions of O4 detection in immunofluorescence assay was indicated with blue boxes.*


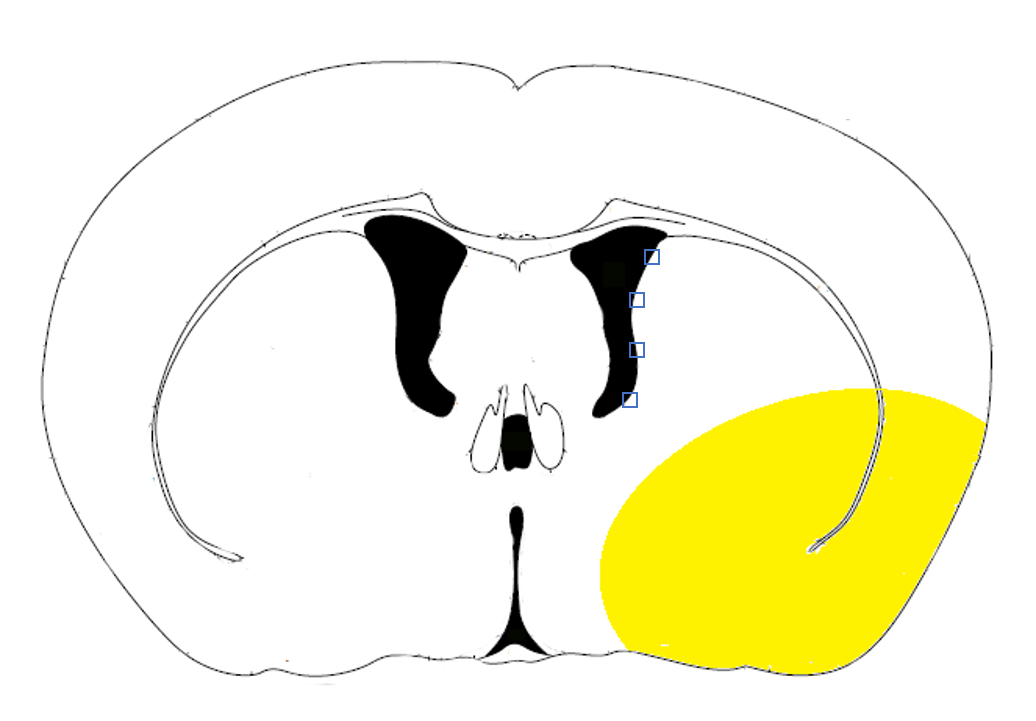


*Supplementary figure 2. Sampling region of MBP detection in immunofluorescence assay was indicated with blue box.*


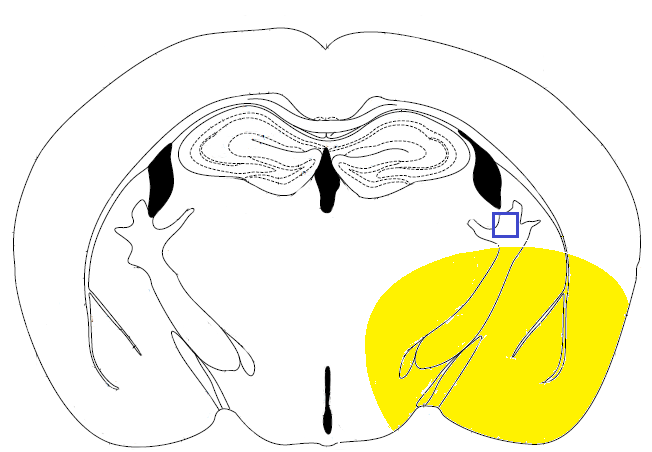

Supplement: Supplementary file 2 [file 7019803.f2.docx]
